# Long non-coding RNA FOXP4-AS1 facilitates the biological functions of hepatocellular carcinoma cells via downregulating ZC3H12D by mediating H3K27me3 through recruitment of EZH2

**DOI:** 10.1007/s10565-021-09642-9

**Published:** 2021-09-21

**Authors:** Junfeng Ye, Yu Fu, Zhongfeng Wang, Jinhai Yu

**Affiliations:** 1 Department of Hepato-Biliary-Pancreatic Surgery, First Hospital, Jilin University, Changchun, 130021 Jilin People’s Republic of China; 2 Department of Hepatology, First Hospital, Jilin University, Changchun, 130021 Jilin People’s Republic of China; 3 Department of Gastrointestinal Surgery, First Hospital, Jilin University, No.71 Xinmin Street, Changchun, 130021 Jilin People’s Republic of China

**Keywords:** Hepatocellular carcinoma, Forkhead box P4 antisense RNA 1, Enhancer of zeste homolog 2, H3K27me3, ZC3H12D, Proliferation, Apoptosis, Migration

## Abstract

**Background:**

Some studies have reported the effect of long non-coding RNA forkhead box P4 antisense RNA 1 (lncRNA FOXP4-AS1) on hepatocellular carcinoma (HCC). Here, we aimed to discuss the effects of FOXP4-AS1/enhancer of zeste homolog 2 (EZH2)/trimethylation of lysine 27 on histone H3 (H3K27me3)/zinc finger CCCH-type containing 12D (ZC3H12D) axis on HCC.

**Methods:**

The expression of FOXP4-AS1, EZH2, and ZC3H12D, and abundance of H3K27me3 in HCC tissues and cells were tested. The relationship between FOXP4-AS1 expression and prognosis of HCC patients was analyzed. The biological functions of HCC cells were detected via loss- and gain-of-function assays. The tumor weight and volume in vivo were tested. The interaction between FOXP4-AS1 and EZH2 as well as that between EZH2 and H3K27me3 was verified.

**Results:**

FOXP4-AS1 and EZH2 expression and H3K27me3 abundance were enhanced while ZC3H12D expression was depressed in HCC tissues and cells. Knockdown of FOXP4-AS1 suppressed biological functions of HCC cells as well as the weight and volume of HCC transplanted tumor. Depleting ZC3H12D reversed the effect of downregulated FOXP4-AS1 on HCC cells. FOXP4-AS1 suppressed ZC3H12D expression via mediating H3K27me3 by recruitment of EZH2.

**Conclusion:**

The key findings of the present study demonstrate that FOXP4-AS1 suppresses ZC3H12D expression via mediating H3K27me3 by recruitment of EZH2, thus promoting the progression of HCC.

**Graphical abstract:**

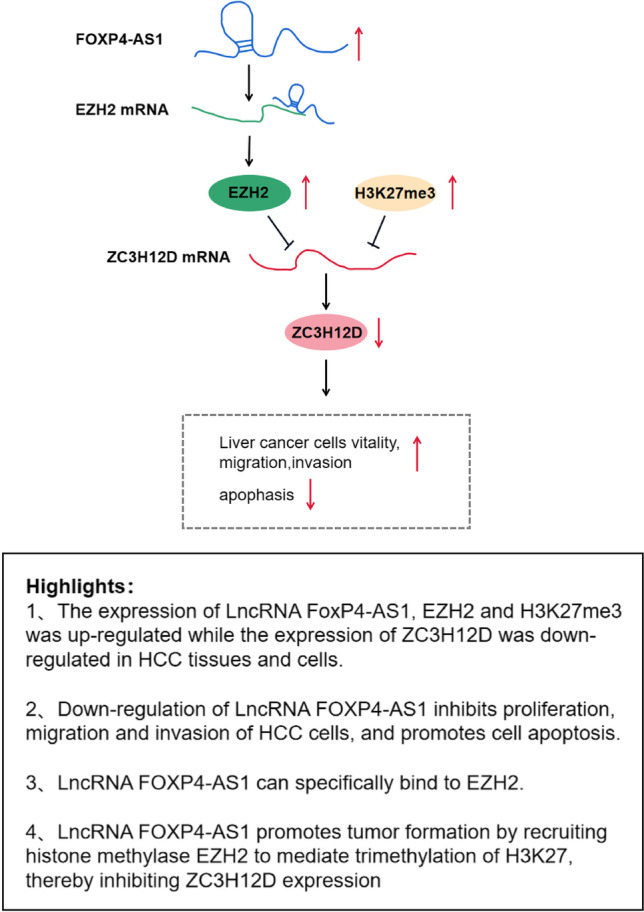

**Supplementary Information:**

The online version contains supplementary material available at 10.1007/s10565-021-09642-9.

## Introduction

Hepatocellular carcinoma (HCC) is one of the prevailing causes of cancer-related mortality in the world (Yang et al. 2019a, b). The high-risk factors of HCC include alcohol abuse, hepatitis B virus or hepatitis C virus infection, and hemochromatosis psychosis (Xu et al. 2016). At present, the combination of computed tomography and serum alpha-fetoprotein (AFP) ultrasounds surveillance is the general strategy for detection and screening of HCC in high-risk population (Shen et al. 2018). Surgery, interventional therapy, radiotherapy, and liver transplantation are therapies that can treat patients with HCC (Que et al. 2018). HCC often results in poor prognosis, and even with optimal treatments, the recurrence rates linked to early and mid-term HCC are high (Yoon et al. 2018). The 5-year survival rate of HCC is only 60%, and the incidence of HCC has increased year by year recently Huang et al. (2019). The tough situation of HCC treatment makes it necessary to further explore the mechanism and find a new therapeutic strategy.

Long non-coding RNA (lncRNA) plays an important role in tumorigenesis, development, tumor metastasis, and prognosis Liu et al. (2020a). Multiple studies have reported that lncRNA forkhead box P4 antisense RNA 1 (FOXP4-AS1) is involved in human cancers, such as colorectal cancer (CRC), gastric cancer (GC), and prostate cancer (PCa) Li et al. 2017a, Chen et al. 2019, Wu et al. 2019). Importantly, it has been revealed that overexpression of FOXP4-AS1 is implicated in HCC (Wang et al. 2019). A study has displayed that FOXP4-AS1 binds to enhancer of zeste homolog 2 (EZH2) and then is involved in the progression of osteosarcoma (Yang et al. 2018). EZH2 is a histone-lysine N-methyltransferase enzyme (Liu, Yang et al. 2020a, b) which has been revealed to participate in human HCC (Huang et al. 2018). A previous study has reported that EZH2 accelerates the progression of HCC (Chen et al. 2018). Trimethylation of lysine 27 on histone H3 (H3K27me3) is a transcription-repressive histone mark which is methylated by EZH2 (Cai et al. 2011). A study has proffered that H3K27me3 can be a powerful diagnostic biomarker for HCC (Gao et al. 2014). It has also been validated that promoter methylation and H3K27 deacetylation are implicated in HCC (Lu et al. 2019). The zinc finger CCCH-type containing 12D (ZC3H12D) is a member of the CCCH-type zinc finger with protein family and reduces inflammation responses through suppressing mRNA stability of proinflammatory genes Zhang et al. (2015). But the relation between ZC3H12D and HCC remains largely unknown. In this study, the aim was to explore the impact of FOXP4-AS1/EZH2/H3K27me3/ZC3H12D axis on HCC.

## Materials and methods

### Ethics statement

The study was approved by the Institutional Review Board of First Hospital, Jilin University. All participants signed a document of informed consent. All animal experiments were in line with the Guide for the Care and Use of Laboratory Animal by International Committees. The protocol was approved by the Institutional Animal Care Use Committee of First Hospital, Jilin University.

### Study subjects

From May 2015 to March 2019, tissue samples of HCC patients treated with hepatobiliary surgery in First Hospital, Jilin University were selected, including 116 cases of HCC tissues and 116 cases of adjacent tissues (normal liver tissue > 2 cm from the edge of neoplastic foci). HCC tissue samples were diagnosed as HCC by pathophysiology, and no tumor cells were identified by pathology in normal adjacent tissues. Freshly isolated samples were taken during the operation and frozen in liquid nitrogen at − 80 °C for reverse transcription quantitative polymerase chain reaction (RT-qPCR) and Western blot assay.

Among the 116 cases, 84 cases were men and 32 cases were women, ranging from 24 to 71 years of age (median age of 53 years), while 88 cases aged less than 60 years and 28 cases aged more than 60 years. According to the degree of tumor differentiation, there were 37 cases with high differentiation and 79 cases with middle and poor differentiation. The tumor node metastasis stage of HCC was in accordance with the standards of International Union Against Cancer, there were 74 cases in the I–II stage and 42 cases in the III–IV stage. No patients treated with radiotherapy and chemotherapy before operation.

### Cell culture

Human HCC cells MHCC-97H, HepG2, LM3, and SMMC-7721 were cultured in high-glucose Dulbecco’s modified eagle medium containing 10% fetal bovine serum (FBS). Human normal liver cell L-02 was cultured in Roswell Park Memorial Institute (RPMI)-1640 medium containing 10% FBS. All cells were cultured in an incubator with 5% CO_2_ and saturated humidity. The cell growth was observed under an inverted microscope, and the medium was renewed every 1–2 days. When reached 80% confluence, cells were detached by trypsin containing ethylene diamine tetraacetic acid and sub-cultured. Cells in well growth were used in experiment.

Human HCC cells MHCC-97H, HepG2, LM3, and SMMC-7721 as well as human normal liver cell L-02 were bought from American Type Culture Collection (VA, USA).

### Cell grouping and transfection

SMMC-7721 cells and LM3 cells were grouped into short hairpin RNA (sh)-negative control (NC) group (cells were transfected with FOXP4-AS1 interference sequence NC), sh-FOXP4-AS1-1 group (cells were transfected with sh-FOXP4-AS1-1 sequence), sh-FOXP4-AS1-2 group (cells were transfected with sh-FOXP4-AS1-2 sequence), pcDNA group (cells were transfected with empty pcDNA3.1 vector), pcDNA-FOXP4-AS1 group (cells were transfected with pcDNA-FOXP4-AS1), sh-FOXP4-AS1-1 + sh-control (CTR) group (cells were transfected with sh-FOXP4-AS1-1 sequence and sh-ZC3H12D CTR), and sh-FOXP4-AS1-1 + sh-ZC3H12D group (cells were transfected with sh-FOXP4-AS1-1 sequence and sh-ZC3H12D).

SMMC-7721 cells and LM3 cells were seeded in a 6-well plate. In the light of the specification of Lipofectamine^TM^2000 transfection reagent (Invitrogen Inc., Carlsbad, CA, USA), the sequences were transfected into cells, and cells were gathered after 48 h of transfection for cell function experiments.

The pcDNA-FOXP4-AS1 and pcDNA3.1 vector (sub-cloned with FOXP4-AS1 and used as a NC), sh-FOXP4-AS1 vector and its NC, as well as sh-ZC3H12D vector and its NC were designed and synthesized by GenePharma (Shanghai, China).

### Cell counting kit–8 assay

Cells were diluted to cell suspension (1.0 × 10^4^ cells/mL) and then seeded in the 96-well plate. Five parallel wells were added in each group. Cell suspension (100 μL) was added into each well of a total of 7 plates. CCK-8 reagent was supplemented into each well at 24 h, 48 h, and 72 h, severally, and then incubated in a 5% CO_2_ incubator for 2 h. The absorbance (*A*) value was gauged by a microplate reader at 450 nm, and the growth curve was drawn.

### Colony formation assay

Cells were cultured in a 6-well plate with suitable density. When the cells adhered to wall, they were cultured for another 10–14 days, then the medium was discarded. Cells were fixed with methanol for 5 min and dyed for 30 min with Giemsa staining solution (Sigma-Aldrich, SF, CA, USA). The colony number with more than 50 cells was counted under the microscope.

### Flow cytometry

Cells were detached, centrifuged, and washed by PBS. The cell concentration was set to 1 × 10^6^ cells/mL. Cells (200 μL) were rinsed for twice with 1 mL pre-cooled phosphate-buffered saline (PBS), centrifuged, suspended, in 100 μL binding buffer, and added with 2 μL Annexin-V-fluorescein isothiocyanate (20 μg/mL). Then, the cells were placed on the ice for 15 min, transferred to the flow tube, and added with 300 μL PBS. Each sample was added with 1 μL propidium iodide (PI) (50 μg/mL) and tested on the flow cytometer. Annexin-V was used as abscissa axis and PI as longitudinal axis; left upper quadrant was mechanical injury cells; right upper quadrant was non-viable apoptotic cells or necrotic cells; left lower quadrant was negative normal cells; right lower quadrant was early apoptotic cells.

### Transwell assay

The matrigel (BD Biosciences, Franklin Lakes, NJ, USA) was dissolved at 4 °C, diluted on the ice by serum-free medium and added into the apical chamber of the Transwell chamber (Corning, N.Y., USA) to evenly cover the bottom of the chamber, then dried overnight. In the cell migration experiment, the apical camber was not coated with matrigel. Cells were detached by trypsin and suspended with serum-free medium. Cell suspension (200 μL) was added into the apical chamber with 1.5 × 10^5^ cells/chamber, and the basolateral chamber was added with 600 μL RPMI-1640 medium supplemented with FBS (100 g/L), then the chamber was cultured for 48 h in an incubator. Afterwards, cells in the apical chamber were wiped off by cotton swabs, cleaned by PBS, and dyed with crystal violet staining solution (1 g/L). Five fields of view were randomly selected to observe the trans-membrane cells by an inverted microscope.

### Tumor xenografts in nude mice

BALB/c mice aged 4 weeks and weighed 16–18 g (male and female) were fed in the specific pathogen-free grade animal room. Cells in the logarithmic phase were amassed and counted, and the cell concentration was set to 5 × 10^7^ cells/mL. Each mouse was subcutaneously injected with 0.1 mL cell suspension at axillary. The survival of mice and the tumor size were observed every day. SMMC-7721 cells and LM3 cells were grouped into (*n* = 6) sh-NC group, sh-FOXP4-AS1-1 group, sh-FOXP4-AS1-2 group, pcDNA group, pcDNA-FOXP4-AS1 group, sh-FOXP4-AS1-1 + sh-CTR group, and sh-FOXP4-AS1-1 + sh-ZC3H12D group. Mice were euthanized at the 21^st^ day. The tumor tissues were collected, then the long diameter (*a*) and the short diameter (*b*) of tumor were measured, the tumor volume (*V*) was counted. *V* (mm^3^) = 1/2 × *a* × *b*^2^. The tumor was weighed and photographed. The tumor tissues were fixed in 4% paraformaldehyde, dehydrated, embedded, and sliced to 4 μm for follow-up experiment.

### Immunohistochemistry

The tissue slices were dewaxed, blocked with 3% hydrogen peroxide for 15 min, cleaned three times with PBS, sealed by 10% normal goat serum for 30 min, then incubated with primary antibody Ki-67 (1: 5000, Abcam Inc., Cambridge, MA, USA) overnight. Next, slices were rinsed three times by PBS with 5 min per time, incubated with secondary antibody for 1 h, developed by diaminobenzidine, then placed in the distilled water. Then, slices were dehydrated by gradient alcohol, cleared by xylene, and sealed by neutral gum. The expression of Ki-67 was analyzed by calculating the percentage of positive cells. Ten fields of view were randomly selected under the microscope, and the percentage of positive cells in total cells was reckoned.

### Terminal deoxynucleotidyl transferase-mediated deoxyuridine triphosphate-biotin nick end labeling staining

The paraffin-embedded tumor tissue sections were mounted and baked, then the apoptotic cells were observed and calculated under the microscope with reference to the instruction of TUNEL cell apoptosis detection kit (Beyotime Institute of Biotechnology, Shanghai, China).

### RT-qPCR

The total RNA in cells and tissues was extracted by Trizol (Invitrogen). The RNA samples of A206/A280 from 1.8 to 2.0 were reversely transcribed. Complementary DNA was synthesized in accordance with reverse transcription PCR with RNA as a template. qPCR was performed with SYBR PrimScript RT-PCR Kit (Takara Bio Inc., Otsu, Shiga, Japan). The target gene expression was analyzed by 2^−ΔΔct^ method. The primers were synthesized by Invitrogen (Table 1).

**Table 1:** Primer sequence

Gene	Sequence
FOXP4-AS1	F: 5′-GTGAGCTTCTGGGTTCGACA-3′
	R: 5′-ATTGAGGGTTAGGGCAGCAC-3′
EZH2	F: 5′-AATCAGAGTACATGCGACT GAGA-3′
	R: 5′- GCTGTATCCTTCGCTGTTTCC-3′
ZC3H12D	F: 5′-AAATATAGTTTGTAGGAGGAAGAGTGTTA-3′
	R: 5′-CAATAAAAAACCACAAAACCATATTATCTC-3′
GAPDH	F: 5′- CACCCACTCCTCCACCTTTG -3′
	R: 5′- CCACCACCCTGTTGCTGTAG-3′

*F*, forward; *R*, reverse; *FOXP4-AS1*, forkhead box P4 antisense RNA 1; *EZH2*, enhancer of zeste homolog 2; *ZC3H12D*, zinc finger CCCH-type containing 12D; *GAPDH*, glyceraldehyde phosphate dehydrogenase

### Chromatin fractionation

Cells were trypsinized, resuspended in the chromatin fractionation buffer (10 mM Hepes [pH = 7.6], 150 mM NaCl, 3 mM MgCl_2_, 0.5%Triton X-100, 1 mM dithiothreitol supplemented with cOmplete™ proteinase inhibitor compound) (Roche, CA, USA) at 1.0 × 10^7^ cells/mL and incubated for 30 min. Precipitated chromatin was centrifuged, and total and chromatin fractionated samples were treated with MNase (S7 Micrococcal nuclease, Roche). All samples were incubated in 1 × Laemmli buffer (10% glycerol, 10 mM Tris [pH = 6.8], 2% sodium dodecyl sulfate [SDS], 0.1 mg/ml bromphenolblue, 2% β-mercaptoethanol) at 95 °C for 5 min and were further analyzed using Western Blot Dalcher et al. (2020).

### Western blot analysis

The total protein was extracted from tissues and cells, and the protein quantification was performed by bovine serum albumin protein kit (Thermo Scientific, Rockford, IL, USA). SDS polyacrylamide gel electrophoresis (10%) was prepared and each well was added with 25 μg protein sample. After electrophoresis, protein was transferred into the polyvinylidene fluoride membrane (Millipore, Bedford, MA, USA), which was blocked by 5% skimmed milk powder for 1 h, washed three times with phosphate-buffered saline with Tween, and incubated with EZH2 antibody (ab191080, 1:1000), H3K27me3 antibody (ab6002, 1: 1000), ZC3H12D antibody (ab100862, 1:1000), histone H3 antibody (ab1791. 1:1000), and glyceraldehyde phosphate dehydrogenase (ab9485, 1:2000) (all from Abcam) overnight, then incubated with secondary antibody labeled by enzyme (Abcam) for 1 h. The membrane was developed and exposed by enhanced chemiluminescence reagent (Pierce Biotechnology, Rockford, IL, USA). The gray value was analyzed by ImageJ software.

### RNA immunoprecipitation assay

Experiment was carried out with reference to the instructions of Magna RIP™ RNA-binding protein immunoprecipitation kit (Millipore). Cells with 80–90% confluence were lysed by RIP lysate. Then, cell extract was incubated with RIP buffer containing magnetic bead which was combined with EZH2 antibody and immunoglobulin G (IgG), respectively. The sample was incubated with protease K and the purified RNA was utilized for RT-qPCR analysis.

### Chromatin immunoprecipitation assay

Cells were prepared with ChIP kit (Millipore) for ChIP detection. Firstly, cells were cross-linked with 1% methanal for 30 min. DNA isolated from cells were smashed to 200–1000 bp by ultrasonication. Then, the DNA fragment was incubated with A/G protein bead containing EZH2 (1:50) and H3K27me3 (1:50, Cell Signaling Technology) or IgG antibody overnight. Next, cells were cleaned, eluted, and de-cross-linked. Finally, the DNA sample was recovered for RT-qPCR detection.

### Statistical analysis

All data were analyzed by SPSS 21.0 software (IBM Corp. Armonk, NY, USA). The measurement data were represented as mean ± standard deviation. Comparisons between two groups were conducted by *t* test while those among multiple groups were assessed by one-way analysis of variance (ANOVA), and the pairwise comparison after ANOVA was analyzed by Tukey’s multiple comparisons test. Pearson analysis was used for correlation analysis, and the prognosis of HCC patients was analyzed by Kaplan–Meier. Statistical significance was set at *P* < 0.05.

## Results

### FOXP4-AS1 and EZH2 expression and H3K27me3 abundance are enhanced while ZC3H12D expression is depressed in HCC tissues and cells

**Fig. 1:**
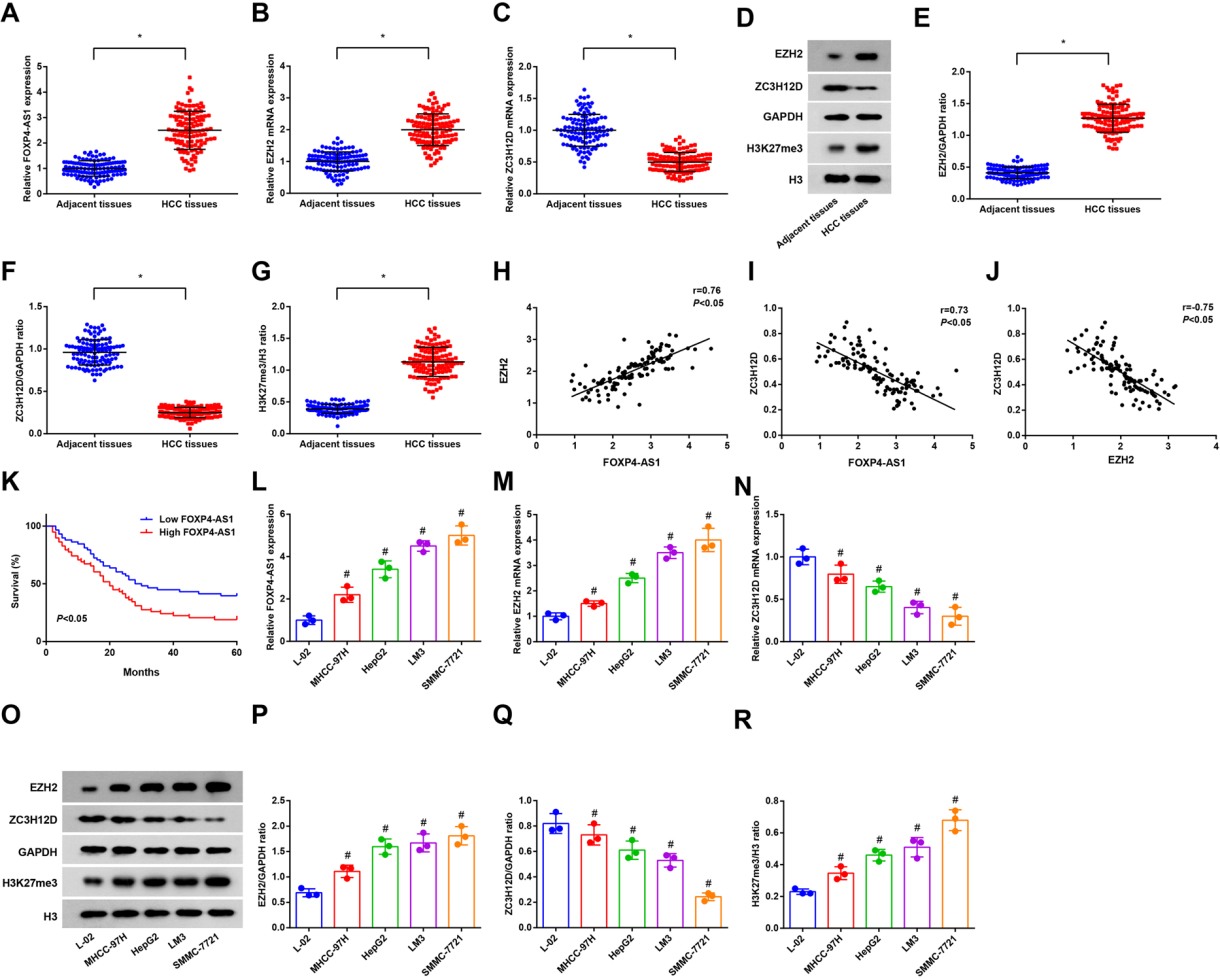
FOXP4-AS1 and EZH2 expression and H3K27me3 abundance are enhanced while ZC3H12D is depressed in HCC tissues and cells. **A**, **B**, **C** FOXP4-AS1 expression, EZH2 mRNA expression and ZC3H12D mRNA expression in HCC tissues and adjacent tissues was determined using RT-qPCR, *n* = 106. **D**, **E**, **F**, **G** Protein bands and protein expression of EZH2, H3K27me3, and ZC3H12D in HCC tissues and adjacent tissues was determined using Western blot analysis, *n* = 106. **H** The correlation between FOXP4-AS1 and EZH2 mRNA expression was analyzed by Pearson analysis, *n* = 106. **I** The correlation between FOXP4-AS1 and ZC3H12D mRNA expression was analyzed by Pearson analysis, *n* = 106. **J** The correlation between EZH2 mRNA and ZC3H12D mRNA expression was analyzed by Pearson analysis, *n* = 106. **K** The effect of FOXP4-AS1 expression on the prognosis of HCC patients was analyzed by Kaplan–Meier survivorship curve, *n* = 106. **L**, **M**, **N** FOXP4-AS1, EZH2, and ZC3H12D mRNA expression in L-02 and HCC cells was determined using RT-qPCR. **O**, **P**, **Q**, **R** Protein bands and protein expression of EZH2, H3K27me3, and ZC3H12D in L-02 and HCC cells was determined using Western blot analysis; *N* = 3. Comparisons between two groups were conducted by *t* test, comparison among multiple groups were assessed by one-way ANOVA followed by Tukey’s multiple comparisons test. Pearson analysis was used for correlation analysis, and the prognosis of HCC patients was analyzed by Kaplan–Meier. ^*^P < 0.05 vs. the adjacent tissues, ^#^*P* < 0.05 vs. the normal liver cells L-02

RT-qPCR and Western blot analysis were used to detect the expression of FOXP4-AS1, EZH2, H3K27me3, and ZC3H12D in HCC tissues and adjacent tissues; it was demonstrated that FOXP4-AS1 and EZH2 expression and H3K27me3 abundance were heightened and ZC3H12D expression was decreased in the HCC tissues versus the adjacent tissues (all *P* < 0.05) (Fig. 1A–G).

Pearson analysis was adopted to analyze the correlation among FOXP4-AS1, EZH2 mRNA, and ZC3H12D mRNA expression. It was suggested that FOXP4-AS1 expression was positively related to EZH2 mRNA expression (Fig. 1H) and was negatively related to ZC3H12D mRNA expression (Fig. 1I) and EZH2 mRNA expression was negatively related to ZC3H12D mRNA expression (Fig. 1J) (all *P* < 0.05).

Kaplan–Meier survival curve was utilized to analyze the effect of FOXP4-AS1 expression on the prognosis of HCC patients, and the results reported that the survival rate of HCC patients in the FOXP4-AS1 low expression group was higher than that in the FOXP4-AS1 high expression group (*P* < 0.05), indicating that the prognosis of HCC patient with low FOXP4-AS1 expression was better (Fig. 1K).

Western blot analysis and RT-qPCR were used to test H3K27me3 abundance and FOXP4-AS1, EZH2, and ZC3H12D expression in human normal liver cells L-02 and human HCC cell lines MHCC-97H, HepG2, LM3, and SMMC-7721. It was displayed that FOXP4-AS1 and EZH2 expression and H3K27me3 abundance were raised and ZC3H12D expression was reduced in HCC cell lines versus L-02 cells (all *P* < 0.05) (Fig. 1L–R).

### Knockdown FOXP4-AS1 suppresses progression of HCC cells as well as the in vivo tumor growth

**Fig. 2:**
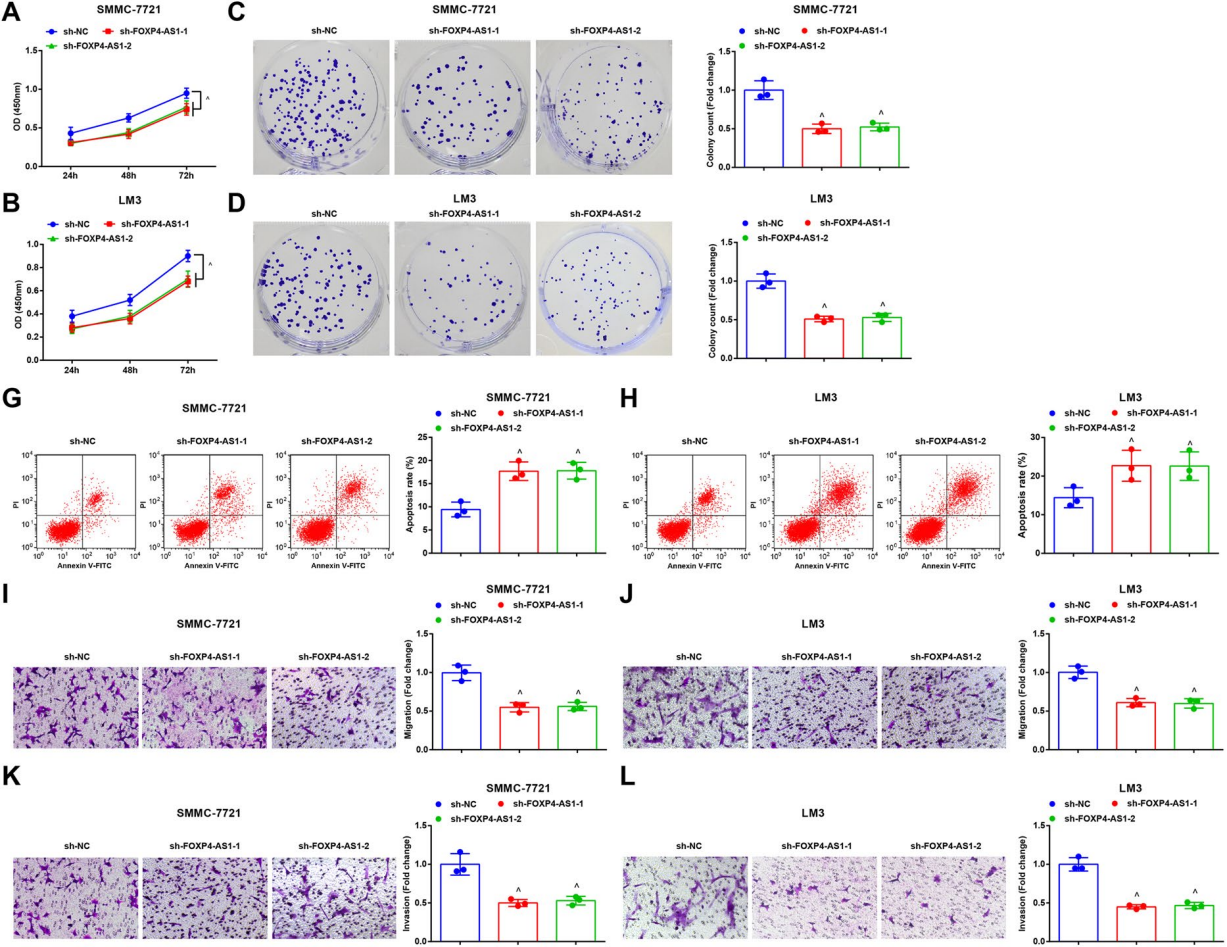
Knockdown FOXP4-AS1 suppresses progression of HCC cells. **A**–**B** The growth curve of SMMC-7721 cells and LM3 cells was tested by CCK-8 assay. **C**–**D** The colony formation ability of SMMC-7721 cells and LM3 cells was tested by colony formation assay. **E**–**F** Cell apoptosis rate of SMMC-7721 cells and LM3 cells was tested by flow cytometry. **G-H** Cell migration ability of SMMC-7721 cells and LM3 cells was tested by Transwell assay. **I-JL** Cell invasion ability of SMMC-7721 cells and LM3 cells was tested by Transwell assay; *N* = 3. Comparisons between two groups were conducted by *t* test, comparison among multiple groups were assessed by one-way ANOVA followed by Tukey’s multiple comparisons test. ^^^*P* < 0.05 vs. the sh-NC group

It was presented by CCK-8 assay, colony formation assay, flow cytometry, and Transwell assay that in the sh-FOXP4-AS1-1 and sh-FOXP4-AS1-2 groups, the proliferation, invasion, colony formation, and migration were markedly reduced, as well as apoptosis was raised versus the sh-NC group (all *P* < 0.05) (Fig. 2A–L).

**Fig. 3:**
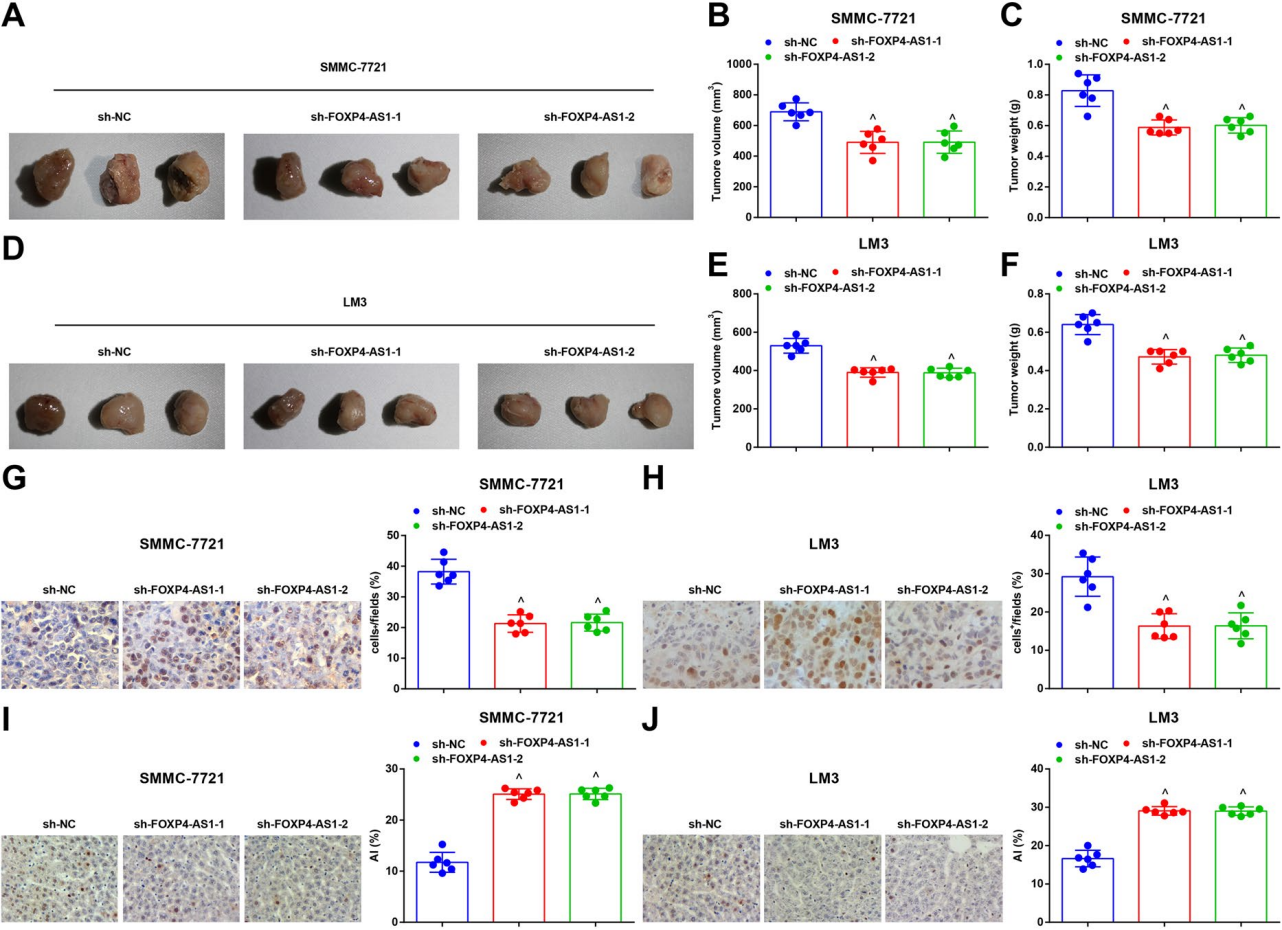
Knockdown FOXP4-AS1 suppresses the in vivo HCC tumor growth. **A&D** Representative tumor images of each group. **B&E** Comparison of tumor volume in each group. **C&F** Comparison of tumor weight in each group. **G**–**H** Ki-67 expression in transplanted tumor tissues was tested by immunohistochemistry (× 400). **I**–**J** Cell apoptosis rate in transplanted tumor tissues was tested by TUNEL staining (× 400); *n* = 6. Comparisons between two groups were conducted by *t* test, comparison among multiple groups were assessed by one-way ANOVA followed by Tukey’s multiple comparisons test. ^^^*P* < 0.05 vs. the sh-NC group

The growth of transplanted tumor of HCC cells after downregulating FOXP4-AS1 was observed. It was displayed that versus the sh-NC group, the weight and volume of tumor were decreased in the sh-FOXP4-AS1-1 and sh-FOXP4-AS1-2 groups (both *P* < 0.05) (Fig. 3A–F). Immunohistochemistry was used to test the expression of Ki-67 in transplanted tumor tissues and it was showed that by comparison with the sh-NC group, Ki-67 expression was reduced in the sh-FOXP4-AS1-1 and sh-FOXP4-AS1-2 group (both *P* < 0.05) (Fig. 3G–H). TUNEL staining results demonstrated that by comparison with the sh-NC group, the apoptosis index of cells in the transplanted tissues was heightened in the sh-FOXP4-AS1-1 and sh-FOXP4-AS1-2 groups (both *P* < 0.05) (Fig. 3I–J).

### Restoring FOXP4-AS1 promotes development of HCC cells as well as the in vivo tumor growth

**Fig. 4:**
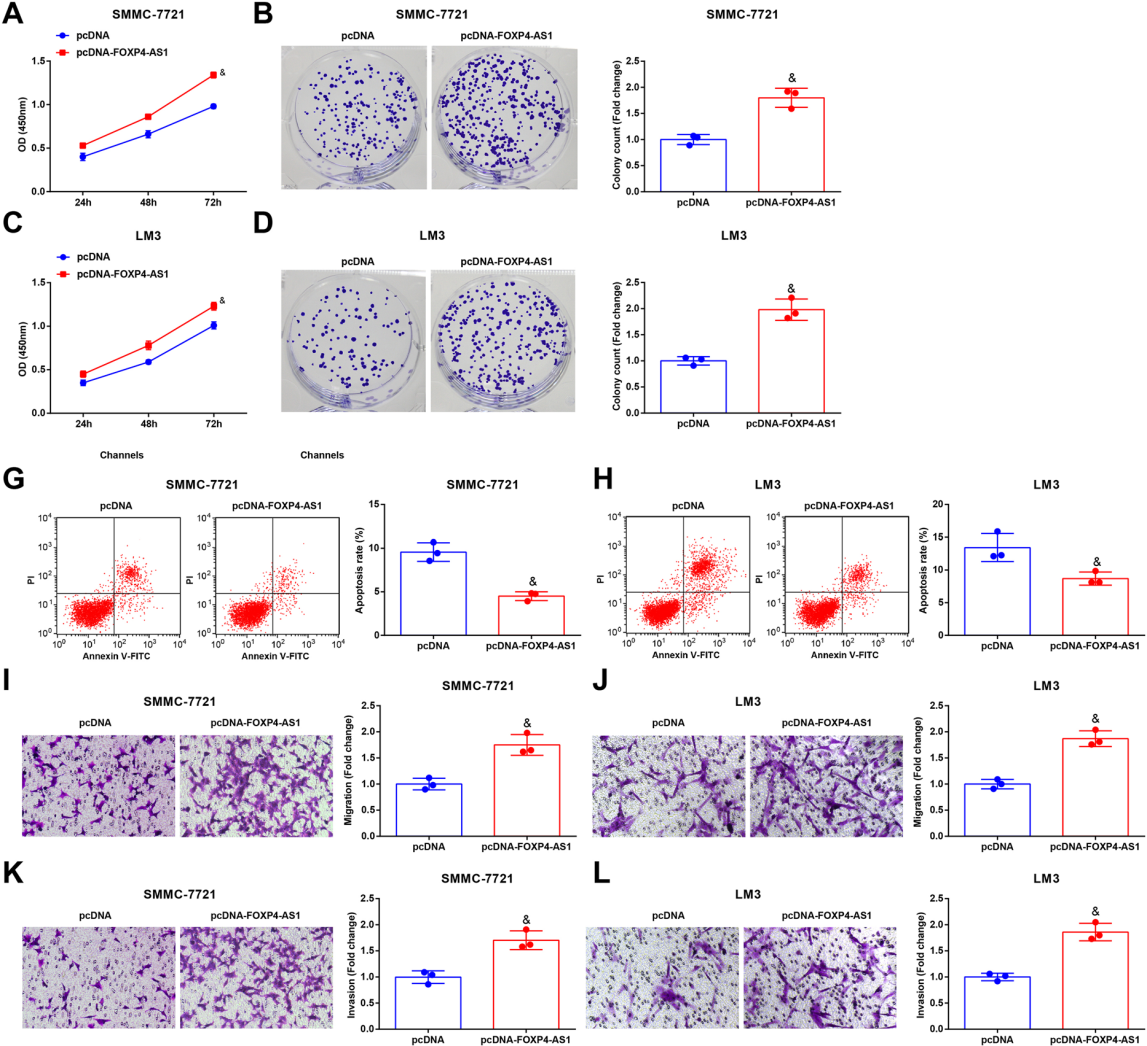
Restoring FOXP4-AS1 promotes development of HCC cells. **A&C** The growth curve of SMMC-7721 cells and LM3 cells was tested by CCK-8 assay. **B**&**D** The colony formation ability of SMMC-7721 cells and LM3 cells was tested by colony formation assay. **E**–**F** Cell apoptosis rate of SMMC-7721 cells and LM3 cells was tested by flow cytometry. **G**–**H** Cell migration ability of SMMC-7721 cells and LM3 cells was tested by Transwell assay. **I**–**J** Cell invasion ability of SMMC-7721 cells and LM3 cells was tested by Transwell assay; *N* = 3. Comparisons between two groups were conducted by *t* test. ^&^*P* < 0.05 vs. the pcDNA group

The investigation focused on function of restored FOXP4-AS1 in HCC reported that in contrast with the pcDNA group, the proliferation, invasion, colony formation, and migration were enhanced, as well as apoptosis were decreased in the pcDNA-FOXP4-AS1 group (all *P* < 0.05) (Fig. 4A–L).

**Fig. 5:**
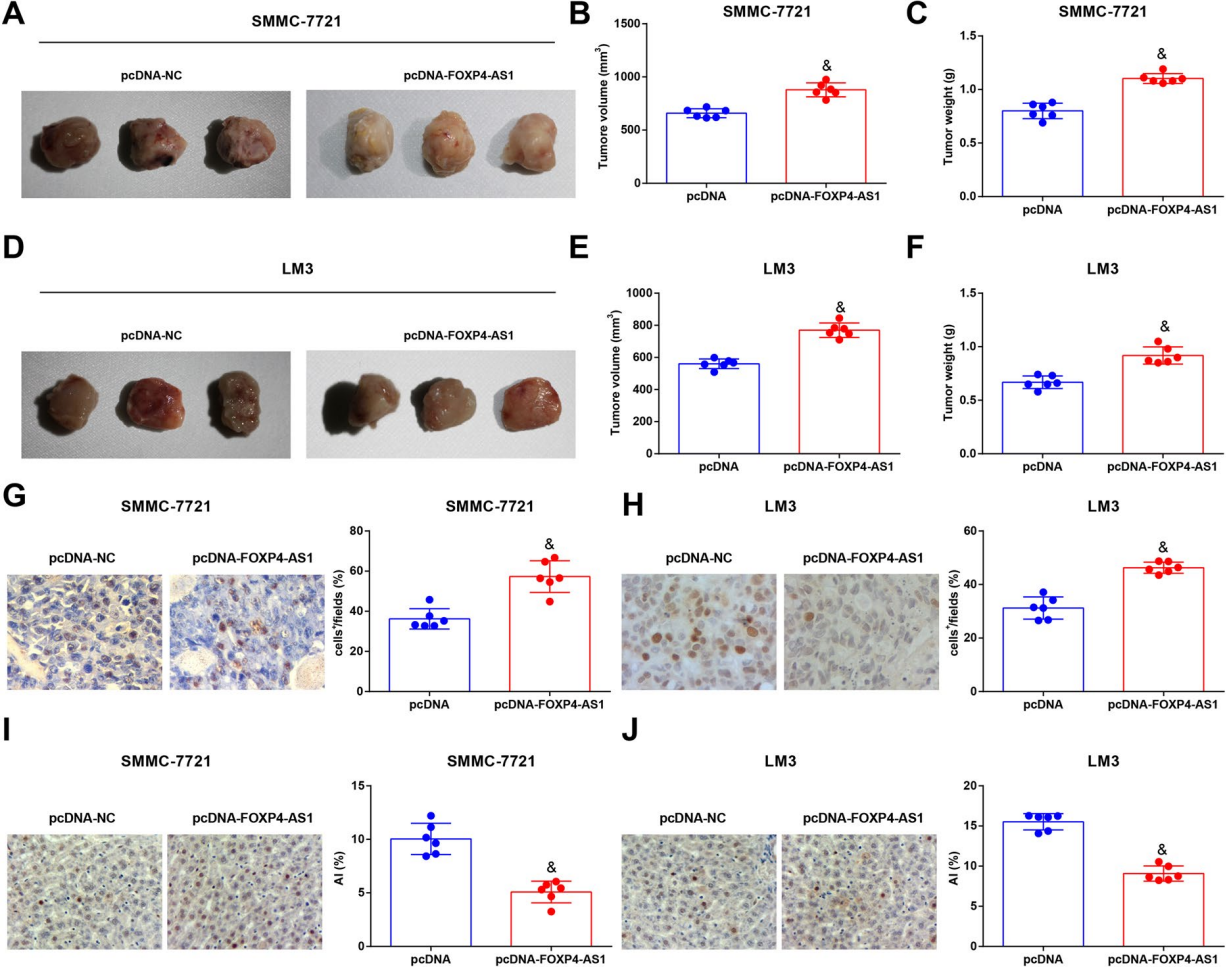
Restoring FOXP4-AS1 promotes the in vivo HCC tumor growth. **A&D** Representative tumor images of each group. **B&E** Comparison of tumor volume in each group. **C&F** Comparison of tumor weight in each group. **G**–**H** Ki-67 expression in transplanted tumor tissues tested by immunohistochemistry (× 400). **I**–**J** Cell apoptosis rate in transplanted tumor tissues tested by TUNEL staining (× 400); *n* = 6. Comparisons between two groups were conducted by *t* test. ^&^*P* < 0.05 vs. the pcDNA group

The growth of transplanted tumor of HCC cells after upregulating FOXP4-AS1 was observed. The results suggested that in contrast with the pcDNA group, the weight and volume of tumor were dramatically enhanced in the pcDNA-FOXP4-AS1 group (*P* < 0.05) (Fig. 5A–F). Immunohistochemistry was used to test the expression of Ki-67 in transplanted tumor tissues and the result demonstrated that versus the pcDNA group, Ki-67 expression was notably raised in the pcDNA-FOXP4-AS1 group (*P* < 0.05) (Fig. 5G–H). TUNEL staining result reported that by comparison with the pcDNA group, the apoptosis index of cells in the transplanted tissues was depressed in the pcDNA-FOXP4-AS1 group (*P* < 0.05) (Fig. 5I–J).

### Depleting ZC3H12D reverses the effect of downregulated FOXP4-AS1 on HCC cells

**Fig. 6:**
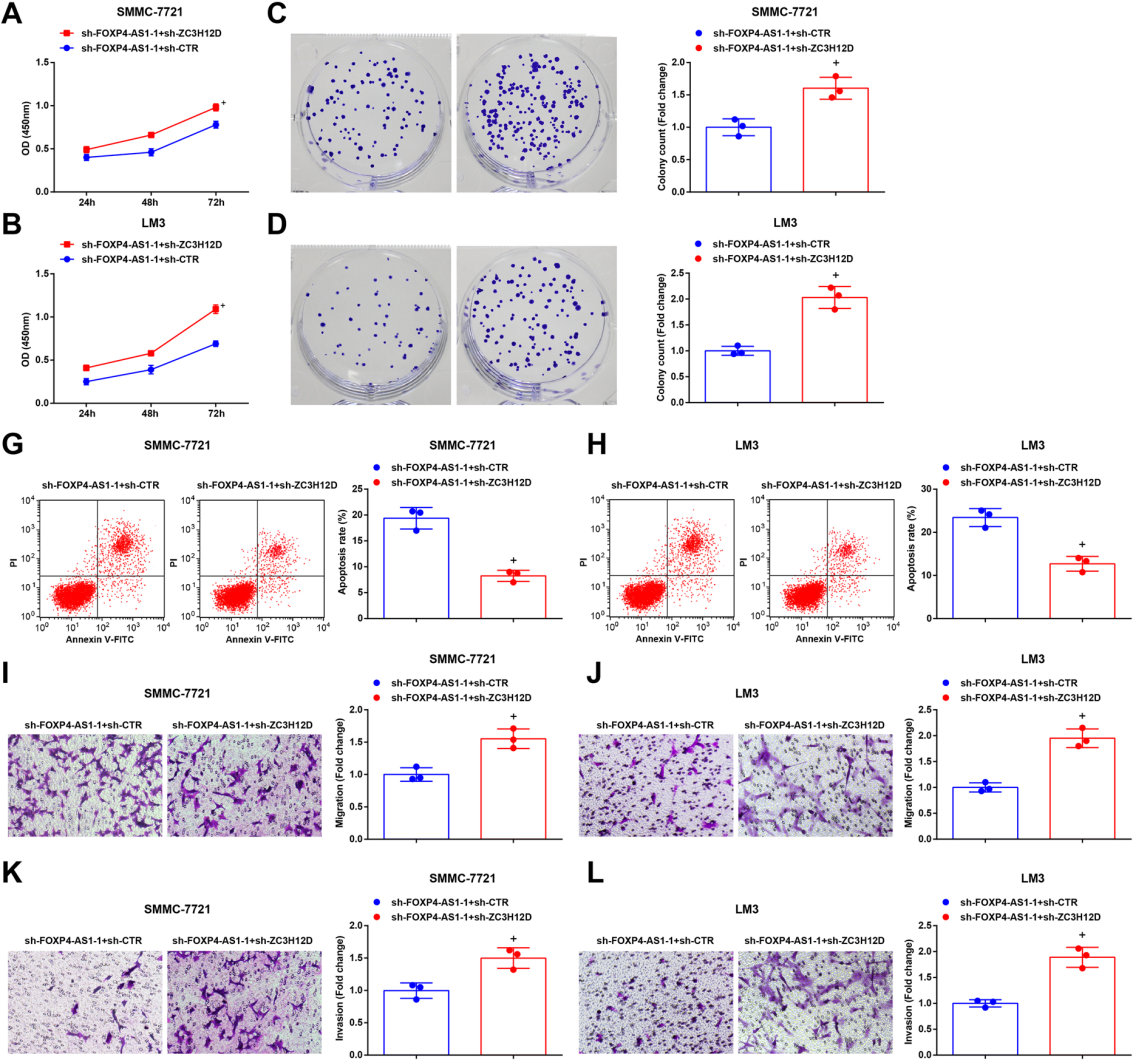
Depleting ZC3H12D reverses the effect of downregulated FOXP4-AS1 on HCC cells. **A**–**B** The growth curve of SMMC-7721 cells and LM3 cells was tested by CCK-8 assay. **C**–**D** The colony formation ability of SMMC-7721 cells and LM3 cells was tested by colony formation assay. **E**–**F** **H**Cell apoptosis rate of SMMC-7721 cells and LM3 cells was tested by flow cytometry. **G**–**H** Cell migration ability of SMMC-7721 cells and LM3 cells was tested by Transwell assay. **I**–**J** Cell invasion ability of SMMC-7721 cells and LM3 cells was tested by Transwell assay; N = 3. Comparisons between two groups were conducted by *t* test. ^+^*P* < 0.05 vs. the sh-FOXP4-AS1-1 + sh-CTR group

In order to discuss the effect of FOXP4-AS1 on the biological functions of HCC cells, some assays were adopted and the results displayed that by comparison with the sh-FOXP4-AS1-1 + sh-CTR group, the proliferation, invasion, colony formation, and migration were elevated, as well as apoptosis were reduced in the sh-FOXP4-AS1-1 + sh-ZC3H12D group (all *P* < 0.05) (Fig. 6A–L).

**Fig. 7:**
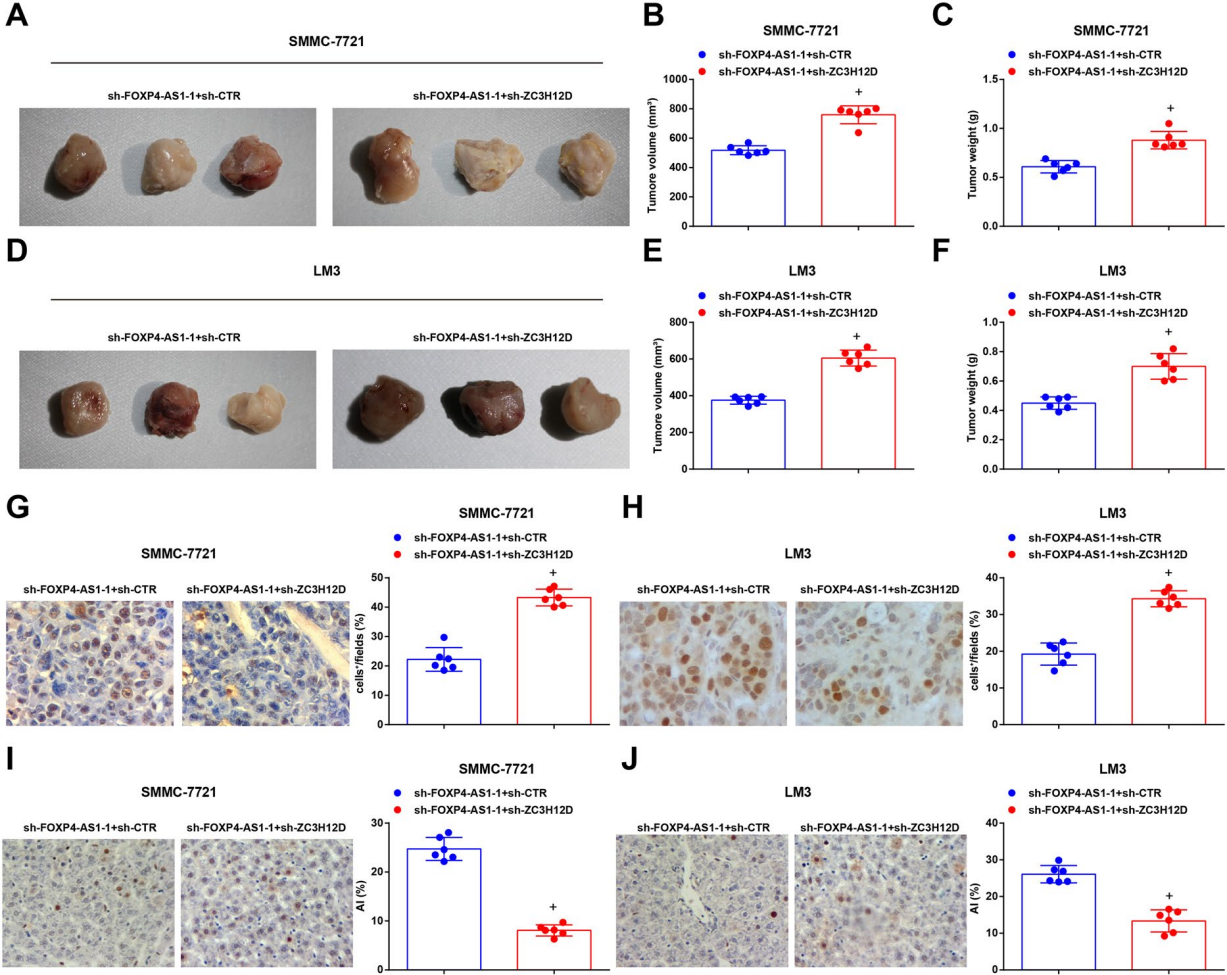
Depleting ZC3H12D reverses the effect of downregulated FOXP4-AS1 on HCC cells. **A**&**D** Representative tumor images of each group. **B&E** Comparison of tumor volume in each group. **C**&**F** Comparison of tumor weight in each group. **G**–**H** Ki-67 expression in transplanted tumor tissues tested by immunohistochemistry (× 400). **I**–**J** Cell apoptosis rate in transplanted tumor tissues tested by TUNEL staining (× 400); *n* = 6. Comparisons between two groups were conducted by *t* test. ^+^*P* < 0.05 vs. the sh-FOXP4-AS1-1 + sh-CTR group

The growth of transplanted tumor of HCC cells was observed by tumor xenografts in nude mice. It was revealed that versus the sh-FOXP4-AS1-1 + sh-CTR group, the weight and volume of tumor were heightened in the sh-FOXP4-AS1-1 + sh-ZC3H12D group (both *P* < 0.05) (Fig. 7A–F). Immunohistochemistry was adopted to detect Ki-67 expression in transplanted tumor tissues and it was reported that versus the sh-FOXP4-AS1-1 + sh-CTR group, Ki-67 expression was elevated in the sh-FOXP4-AS1-1 + sh-ZC3H12D group (*P* < 0.05) (Fig. 7G–H). TUNEL staining result indicated that versus the sh-FOXP4-AS1-1 + sh-CTR group, the apoptosis index of cells in the transplanted tissues was decreased in the sh-FOXP4-AS1-1 + sh-ZC3H12D group (*P* < 0.05) (Fig. 7I–J).

### FOXP4-AS1 binds to EZH2 which combines to the promoter region of ZC3H12D and mediates the methylation of H3K27me3

**Fig. 8:**
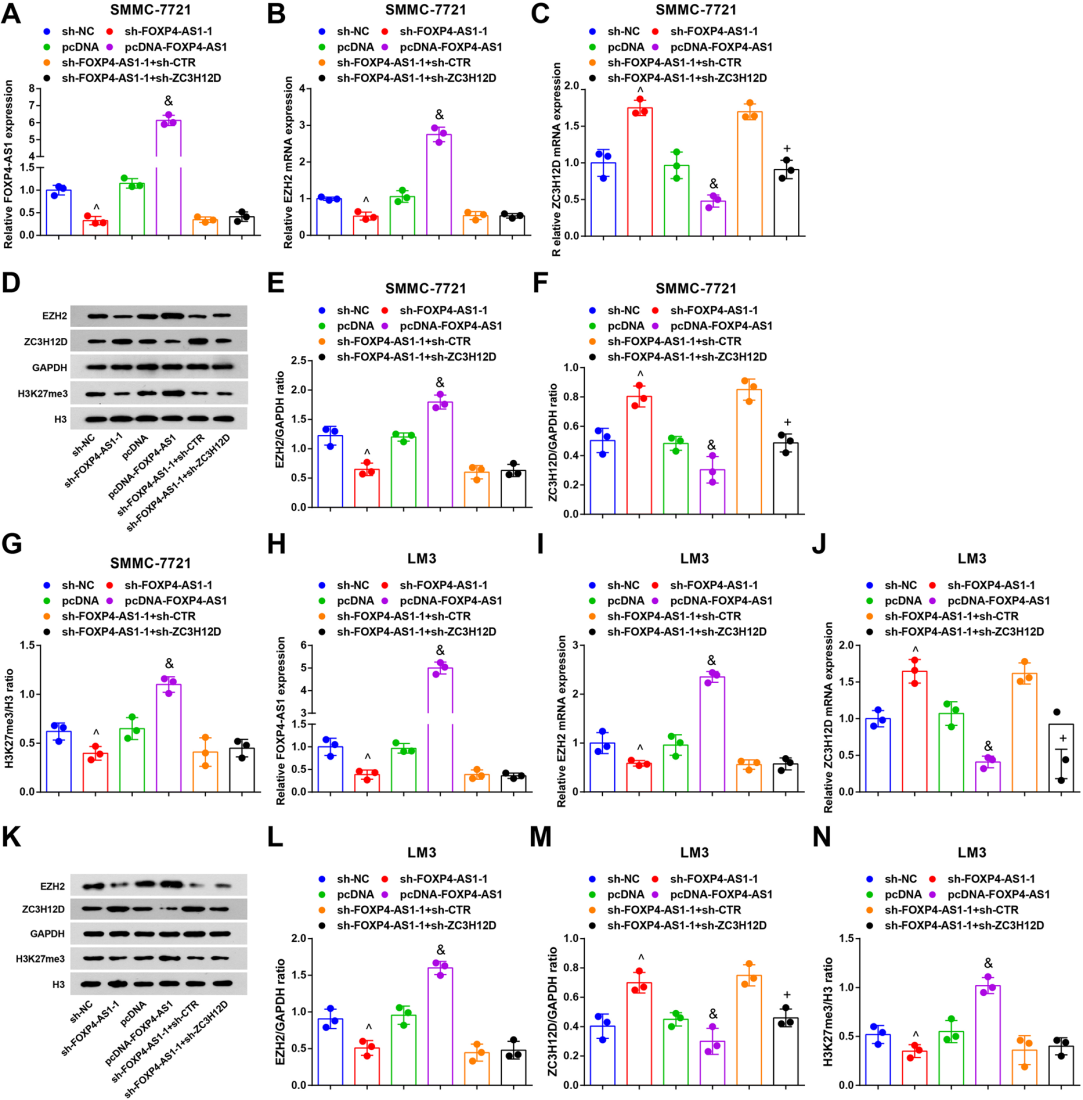
Expression levels of FOXP4-AS1, EZH2, and ZC3H12D in SMMC-7721 cells and LM3 cells. **A**, **B**, **C** FOXP4-AS1, EZH2 mRNA, and ZC3H12D mRNA expression in SMMC-7721 cells was tested by RT-qPCR. **D**, **E**, **F**, **G** Protein bands and protein expression of EZH2, H3K27me3, and ZC3H12D in SMMC-7721 cells were tested by Western blot analysis. **H**, **I**, **J** FOXP4-AS1, EZH2 mRNA, and ZC3H12D mRNA expression in LM3 cells was tested by RT-qPCR. **K**, **L**, **M**, **N** Protein bands and protein expression of EZH2, H3K27me3, and ZC3H12D in LM3 cells were tested by Western blot analysis. *N* = 3. Comparisons between two groups were conducted by *t* test, comparison among multiple groups were assessed by one-way ANOVA followed by Tukey’s multiple comparisons test. ^^^*P* < 0.05 vs. the sh-NC group. ^&^*P* < 0.05 vs. the pcDNA group. ^+^*P* < 0.05 vs. the sh-FOXP4-AS1-1 + sh-CTR group

RT-qPCR and Western blot analysis revealed that in contrast with the sh-NC group, EZH2 expression and H3K27me3 abundance were remarkably decreased while ZC3H12D expression was elevated in the sh-FOXP4-AS1-1 group (all *P* < 0.05). By comparison with the pcDNA group, EZH2 expression and H3K27me3 abundance were enhanced while ZC3H12D expression was reduced in the pcDNA-FOXP4-AS1 group (all *P* < 0.05) (Fig. 8A–N).

**Fig. 9:**
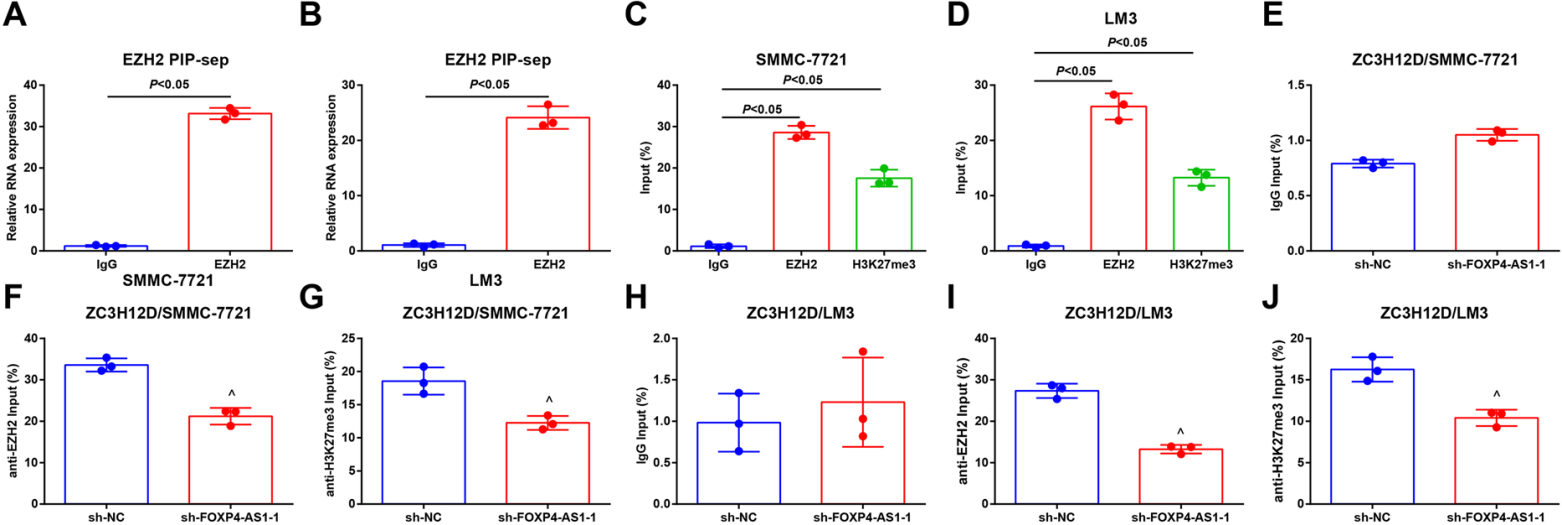
FOXP4-AS1 binds to EZH2 which combines to the promoter region of ZC3H12D and mediates the methylation of H3K27me3. **A**–**B** The potential binding protein of FOXP4-AS1 was detected by RIP assay. **C**–**D** The binding of EZH2 and H3K27me3 in the promoter region of ZC3H12D in SMMC-7721 and LM3 cells was tested by ChIP assay. **E**, **F**, **G**, **H**, **I**, **J** The binding of EZH2 and H3K27me3 in the promoter region of ZC3H12D after downregulated FOXP4-AS1 in SMMC-7721 and LM3 cells was tested by ChIP assay. IgG was the loading control; *N* = 3. Comparisons between two groups were conducted by *t* test, comparison among multiple groups were assessed by one-way ANOVA followed by Tukey’s multiple comparisons test. ^^^*P* < 0.05 vs. the sh-NC group. ^&^*P* < 0.05 vs. the pcDNA group. ^+^*P* < 0.05 vs. the sh-FOXP4-AS1-1 + sh-CTR group

In general, lncRNA played a role in tumor development process through binding to corresponding RNA-binding proteins. Thus, the potential target binding proteins of FOXP4-AS1 were tested by RIP assay, and it was reported that FOXP4-AS1 could bind to EZH2 (Fig. 9A,B). ChIP assay was used to further validate the binding of FOXP4-AS1 and ZC3H12D. The results implied that in HCC cells, EZH2 and H3K27me3 were enriched in ZC3H12D promoter region, and the enrichment was inhibited by FOXP4-AS1 silencing (Fig. 9C–J). The data suggested that FOXP4-AS1 recruits EZH2 to the promoter region of ZC3H12D to mediate H3K27me3 methylation level, thus inhibiting ZC3H12D expression.

## Discussion

HCC refers to the most common primary liver tumor globally (Santopaolo et al. 2019). In a study conducted by Wang et al., it has been shown that FOXP4-AS1 level is related to size of tumor, serum AFP, serum aspartate aminotransferase, liver cirrhosis and patient age in HCC samples (Wang et al. 2019). Also, a recent study has provided a proof that depletion of EZH2 suppresses cell proliferation of HCC (Li, Li et al. 2017a, b). It is customarily considered that EZH2-mediated H3K27me3 is implicated in HCC (Li et al. 2016). It is presented that the anti-tumor properties of ZC3H12D are further confirmed and its expression promotes apoptosis, represses Rb phosphorylation, and cellular growth in Jurkat cells (Wawro et al. 2017). Based on these facts, the study aimed to explore the effect of FOXP4-AS1/EZH2/H3K27me3/ZC3H12D axis on HCC.

Of particular interest was the fact that in our study, the expression of FOXP4-AS1 and EZH2, and abundance of H3K27me3 were raised while ZC3H12D expression was decreased in HCC tissues and cells. A study has presented that the expression of FOXP4-AS1 is heightened in CRC tissues and cell lines while its upregulation positively linked to larger tumor size and advanced pathological stages Li et al. (2017a). Moreover, FOXP4-AS1 expression is also raised in GC tissues and PCa tissues (Chen et al. 2019; Wu et al. 2019). Another study has purported that FOXP4-AS1 expression is markedly heightened in HCC samples versus the controls (Wang et al. 2019). As for the abnormal expression of EZH2, a study has presented that the expression of EZH2 is enhanced in most of HCC tissues and all cell lines (Xu et al. 2014). It has been displayed that EZH2 expression is elevated in HCC (Yang et al. 2019a, b). A study also demonstrated that EZH2 expression and H3K27me3 abundance are concomitantly raised in human HCC by comparison with non-tumor livers (Gnani et al. 2017). In a study conducted by Gao et al., it has been shown that H3K27me3 is up-regulated in HCC samples at the promoters of certain target (Gao et al. 2014). Moreover, it has been displayed that ZC3H12D is dramatically downregulated in airway immune cells during acute lung injury induced by lipopolysaccharides Zhang et al. (2015). All these evidences confirmed that the expression of FOXP4-AS1 and EZH2 expression and H3K27me3 abundance were raised while ZC3H12D expression was inhibited in HCC tissues and cells, but the expression of ZC3H12D in HCC cells and tissues needs further investigation.

In addition, our study reported that FOXP4-AS1 could directly bind to EZH2, and EZH2 could bind to the promoter region of ZC3H12D and mediate the methylation of H3K27me3. A study has revealed that FOXP4-AS1 is involved in the progression of osteosarcoma through binding to EZH2 (Yang et al. 2018). It has been indicated that FOXP4-AS1 can bind to EZH2, and positively modulated its expression level (Chen et al. 2019). A study has contended that EZH2 and H3K27me3 are enriched in methylated HCC cell lines (Wei, Liu et al. 2020a, b). Another study has revealed that EZH2 and H3K27me3 are moderately enriched in HCC cells as well as EZH2-mediated H3K27me3 contributes to the suppression of miR-22 transcriptional in a DNA hyper-methylation-independent manner (Chen et al. 2018). However, the binding relationship between ZC3H12D and EZH2 needs further study.

Another result emerged from our data indicating that lowly expressed FOXP4-AS1 repressed proliferation, colony formation ability, invasion and migration ability as well as facilitated the apoptosis of HCC cells. Moreover, this study also demonstrated that downregulated FOXP4-AS1 reduced the weight and volume of tumor, also raised the apoptosis rate in transplanted tumor tissues of HCC. It has been suggested previously that depleting FOXP4-AS1 induces acceleration in number of cells in G0/G1 phase and inhibition in number of cells in S-phase in CRC, also, represses tumor growth in vivo Li et al. (2017a). It is reported that downregulation of FOXP4-AS1 reduces cell proliferation and facilitates cell apoptosis, suggesting that FOXP4-AS1 presents oncogenic functions in PCa tumorigenesis (Wu et al. 2019). Another study has verified that lowly expressed FOXP4-AS1 results in reduction of proliferation, migration and invasion, and promotes cell cycle in osteosarcoma cells (Yang et al. 2018). Furthermore, a result emerged from our study revealed that depleting ZC3H12D reversed the effect of downregulated FOXP4-AS1 on HCC cells, which needed further validation in the future.

## Conclusion

As discussed above, it is discovered that lncRNA FOXP4-AS1 suppresses ZC3H12D expression via mediating H3K27me3 by recruitment of EZH2, thus promoting the progression of HCC. However, a conclusion about the effects of FOXP4-AS1 and ZC3H12D cannot be made clearly due to limited known researches on this. There are many genes that may be involved in mediating H3K27me3 and EZH2, which should be explored further in the later research.

## Supplementary Information

Below is the link to the electronic supplementary material.

Supplementary file1 (JPG 2667 KB)

## Abbreviations

lncRNA FOXP4-AS1: Long non-coding RNA forkhead box P4 antisense RNA 1
HCC: Hepatocellular carcinoma
EZH2: Enhancer of zeste homolog 2
HCC: Hepatocellular carcinoma
AFP: Alpha-fetoprotein
lncRNA: Long non-coding RNA
PCa: Prostate cancer
RT-qPCR: Reverse transcription quantitative polymerase chain reaction
CCKC: Cell counting kit
PBS: Phosphate-buffered saline
SPF: Specific pathogen-free
DAB: Diaminobenzidine
TUNEL: Triphosphate-biotin nick end labeling
RIPRNA: Immunoprecipitation
ChIP: Chromatin immunoprecipitation
ANOVA: Analysis of variance

## References

[CR1] Cai MY, Hou JH, Rao HL, Luo RZ, Li M, Pei XQ, Lin MC, Guan XY, Kung HF, Zeng YX, Xie D (2011). High expression of H3K27me3 in human hepatocellular carcinomas correlates closely with vascular invasion and predicts worse prognosis in patients. Mol Med.

[CR2] Chen RY, Ju Q, Feng LM, Yuan Q, Zhang L (2019). The carcinogenic complex lncRNA FOXP4-AS1/EZH2/LSD1 accelerates proliferation, migration and invasion of gastric cancer. Eur Rev Med Pharmacol Sci.

[CR3] Chen S, Pu J, Bai J, Yin Y, Wu K, Wang J, Shuai X, Gao J, Tao K, Wang G, Li H (2018). EZH2 promotes hepatocellular carcinoma progression through modulating miR-22/galectin-9 axis. J Exp Clin Cancer Res.

[CR4] Dalcher D, Tan JY, Bersaglieri C, Pena-Hernandez R, Vollenweider E, Zeyen S, Schmid MW, Bianchi V, Butz S, Roganowicz M, Kuzyakiv R, Baubec T, Marques AC, Santoro R (2020). BAZ2A safeguards genome architecture of ground-state pluripotent stem cells. EMBO J.

[CR5] Gao SB, Xu B, Ding LH, Zheng QL, Zhang L, Zheng QF, Li SH, Feng ZJ, Wei J, Yin ZY, Hua X, Jin GH (2014). The functional and mechanistic relatedness of EZH2 and menin in hepatocellular carcinoma. J Hepatol.

[CR6] Gnani D, Romito I, Artuso S, Chierici M, De Stefanis C, Panera N, Crudele A, Ceccarelli S, Carcarino E, D’Oria V, Porru M, Giorda E, Ferrari K, Miele L, Villa E, Balsano C, Pasini D, Furlanello C, Locatelli F, Nobili V, Rota R, Leonetti C, Alisi A. Focal adhesion kinase depletion reduces human hepatocellular carcinoma growth by repressing enhancer of zeste homolog 2. Cell Death Differ. 2017;24(5):889–902. 10.1038/cdd.2017.34 PMC5423113 28338656

[CR7] Huang B, Huang M, Li Q (2018). MiR-137 suppresses migration and invasion by targeting EZH2-STAT3 signaling in human hepatocellular carcinoma. Pathol Res Pract.

[CR8] Huang D, Wei Y, Zhu J, Wang F (2019). Long non-coding RNA SNHG1 functions as a competitive endogenous RNA to regulate PDCD4 expression by sponging miR-195–5p in hepatocellular carcinoma. Gene.

[CR9] Li CP, Cai MY, Jiang LJ, Mai SJ, Chen JW, Wang FW, Liao YJ, Chen WH, Jin XH, Pei XQ, Guan XY, Zeng MS, Xie D (2016). CLDN14 is epigenetically silenced by EZH2-mediated H3K27ME3 and is a novel prognostic biomarker in hepatocellular carcinoma. Carcinogenesis.

[CR10] Li, J., Y. Lian, C. Yan, Z. Cai, J. Ding, Z. Ma, P. Peng and K. Wang (2017). Long non-coding RNA FOXP4-AS1 is an unfavourable prognostic factor and regulates proliferation and apoptosis in colorectal cancer. Cell Prolif **50**(1). 10.1111/cpr.12312 PMC6529074 27790757

[CR11] Li Q, Li B, Dong C, Wang Y, Li Q (2017). 20(S)-Ginsenoside Rh2 suppresses proliferation and migration of hepatocellular carcinoma cells by targeting EZH2 to regulate CDKN2A-2B gene cluster transcription. Eur J Pharmacol.

[CR12] Liu, X. G., H. Xu, M. Chen, X. Y. Tan, X. F. Chen, Y. G. Yang, M. Z. Lin, G. H. Liu, X. L. Liang, Y. B. Qian, G. J. Yuan, M. Q. Chen, W. T. Li, H. L. Miao, M. Y. Li, X. W. Liao, W. Dai and N. P. Chen (2020). Identify potential clinical significance of long noncoding RNA forkhead box P4 antisense RNA 1 in patients with early stage pancreatic ductal adenocarcinoma. Cancer Med. 10.1002/cam4.2818 PMC7064149 31991068

[CR13] Liu Z, Yang L, Zhong C, Zhou L (2020). EZH2 regulates H2B phosphorylation and elevates colon cancer cell autophagy. J Cell Physiol.

[CR14] Lu S, Lu H, Jin R, Mo Z (2019). Promoter methylation and H3K27 deacetylation regulate the transcription of VIPR1 in hepatocellular carcinoma. Biochem Biophys Res Commun.

[CR15] Que KT, Zhou Y, You Y, Zhang Z, Zhao XP, Gong JP, Liu ZJ (2018). MicroRNA-31-5p regulates chemosensitivity by preventing the nuclear location of PARP1 in hepatocellular carcinoma. J Exp Clin Cancer Res.

[CR16] Santopaolo F, Lenci I, Milana M, Manzia TM, Baiocchi L (2019). Liver transplantation for hepatocellular carcinoma: where do we stand?. World J Gastroenterol.

[CR17] Shen X, Xue Y, Cong H, Wang X, Ju S (2018). Dysregulation of serum microRNA-574-3p and its clinical significance in hepatocellular carcinoma. Ann Clin Biochem.

[CR18] Wang D, Bai T, Chen G, Liu J, Chen M, Zhao Y, Luo T, Chen J, Li L, Zhang C, Li H (2019). Upregulation of long non-coding RNA FOXP4-AS1 and its regulatory network in hepatocellular carcinoma. Onco Targets Ther.

[CR19] Wawro M, Kochan J, Krzanik S, Jura J, Kasza A (2017). Intact NYN/PIN-like domain is crucial for the degradation of inflammation-related transcripts by ZC3H12D. J Cell Biochem.

[CR21] Wu X, Xiao Y, Zhou Y, Zhou Z, Yan W (2019). LncRNA FOXP4-AS1 is activated by PAX5 and promotes the growth of prostate cancer by sequestering miR-3184-5p to upregulate FOXP4. Cell Death Dis.

[CR22] Xu J, Li J, Zheng TH, Bai L, Liu ZJ (2016). MicroRNAs in the Occurrence and development of primary hepatocellular carcinoma. Adv Clin Exp Med.

[CR23] Xu L, Beckebaum S, Iacob S, Wu G, Kaiser GM, Radtke A, Liu C, Kabar I, Schmidt HH, Zhang X, Lu M, Cicinnati VR (2014). MicroRNA-101 inhibits human hepatocellular carcinoma progression through EZH2 downregulation and increased cytostatic drug sensitivity. J Hepatol.

[CR24] Yang C, Ma X, Guan G, Liu H, Yang Y, Niu Q, Wu Z, Jiang Y, Bian C, Zang Y, Zhuang L (2019). MicroRNA-766 promotes cancer progression by targeting NR3C2 in hepatocellular carcinoma. FASEB J.

[CR25] Yang L, Ge D, Chen X, Qiu J, Yin Z, Zheng S, Jiang C (2018). FOXP4-AS1 participates in the development and progression of osteosarcoma by downregulating LATS1 via binding to LSD1 and EZH2. Biochem Biophys Res Commun.

[CR26] Yang PM, Hong YH, Hsu KC, Liu TP (2019). p38alpha/S1P/SREBP2 activation by the SAM-competitive EZH2 inhibitor GSK343 limits its anticancer activity but creates a druggable vulnerability in hepatocellular carcinoma. Am J Cancer Res.

[CR27] Yoon JS, Kim G, Lee YR, Park SY, Tak WY, Kweon YO, Park JG, Lee HW, Han YS, Ha HT, Chun JM, Jang SY, Hur K (2018). Clinical significance of microRNA-21 expression in disease progression of patients with hepatocellular carcinoma. Biomark Med.

[CR28] Zhang H, Wang WC, Chen JK, Zhou L, Wang M, Wang ZD, Yang B, Xia YM, Lei S, Fu EQ, Jiang T (2015). ZC3H12D attenuated inflammation responses by reducing mRNA stability of proinflammatory genes. Mol Immunol.

