# Supplementary figures and images for: Long non-coding RNA FOXP4-AS1 facilitates the biological functions of hepatocellular carcinoma cells via downregulating ZC3H12D by mediating H3K27me3 through recruitment of EZH2
Source: Cell Biol Toxicol. 2021 Sep 21;38(6):1047–62. doi: 10.1007/s10565-021-09642-9 (PMC9750913; doi:10.1007/s10565-021-09642-9)

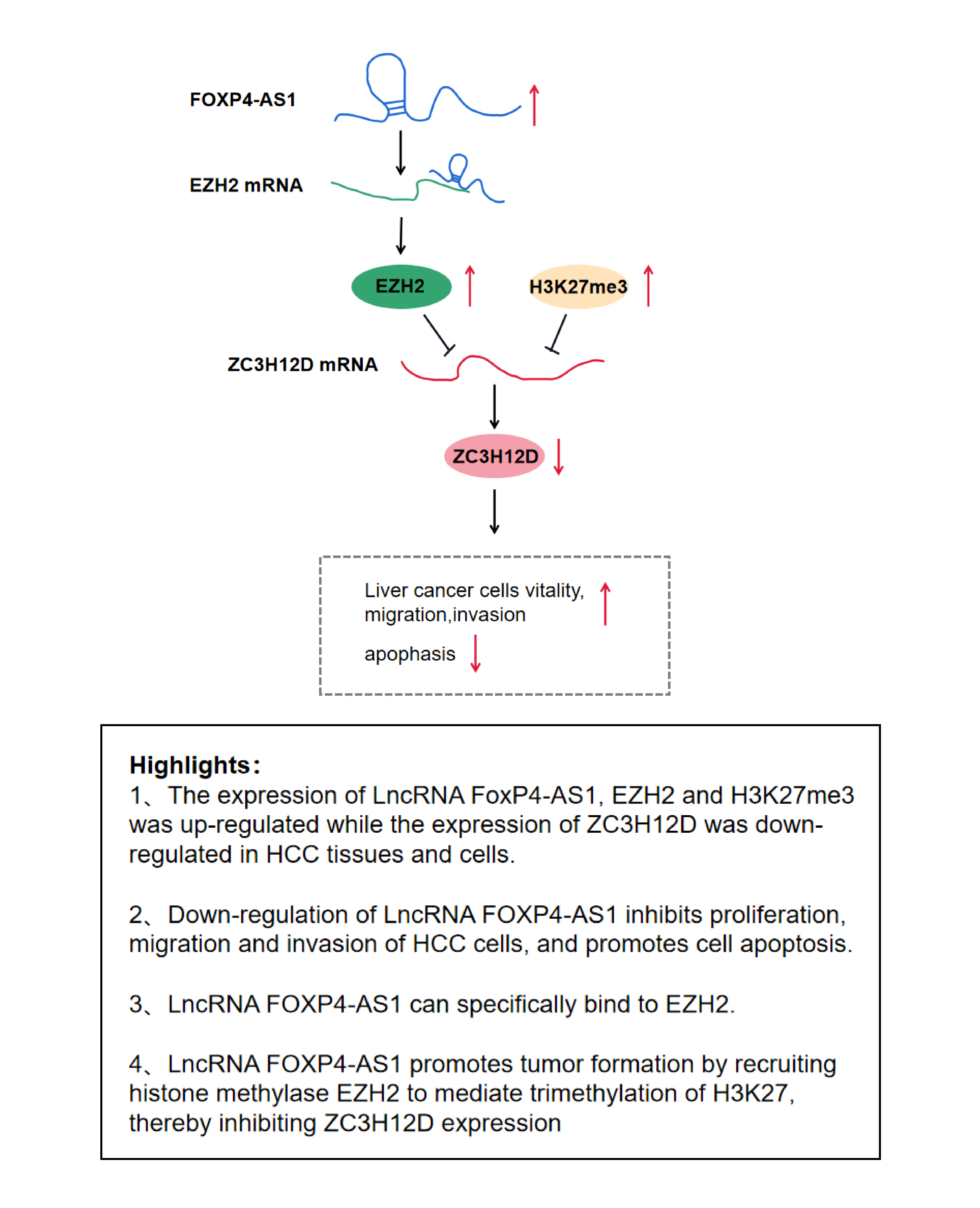

Supplement: Supplementary file 1 — Supplementary file1 (JPG 2667 KB) [file 10565_2021_9642_MOESM1_ESM.jpg]
